# Criminal Responsibility of the Insane

**Published:** 1904-01-09

**Authors:** 


### The Hospital.

A Journal of

The Medical Sciences and Hospital Administration.

Vol. XXXV.—No. 902. JANUARY 9, 1904.

### Criminal Responsibility of the Insane.

The current number of the *Edinburgh Medical Journal* contains the first of a series of papers by Sir John Batty Tuke, M.P., and Mr. Howden, M.A., on the Relations of the Insanities to Criminal Responsibility and Civil Capacity; and the great importance of the subject dealt with, as well as the exceptional fitness of the authors, induces us to call early attention to their work. A note informs us that the papers were originally projected by Sir John in 1887, in collaboration with the late Mr. Alexander Gibson, but were laid aside in consequence of his decease, and have now been resumed with the help of another advocate, whose share of the joint work will be to assist in a general rearrangement, as well as to furnish a supplement, rendered necessary by the lapse of time, to the chapters bearing immediately on the theory and practice of law. In the opening paper we find a masterly analysis of the difficulties by which the questions at issue are surrounded, and of the circumstances by which it has been rendered inevitable that conflicts between law and medicine should occur. The descriptions given of many forms of insanity "seemed perilously like ordinary depravity," and although the expert declared that a difference was visible, and that the cases belonged to a class which he recognised as insanity, "the judge on his side was also an expert, and declared them to belong to familiar forms of crime." We have here, indeed, the problem in a nutshell, and we have also what may be described as a definition of insanity, in a declaration that all cases properly so called result from conditions of physical disease or defect. "What was formerly called mental disease is now known to be the effect and symptom of physical disease. In its widest sense we apply the term insanity to those defects or aberrations of the mental functions which are caused by physical defect or disease of the brain."

Having reached this point, and having dismissed "diseases of the mind" from consideration, it is manifest that two questions of vital importance will remain. Are there any definite symptoms by which the existence of disease of the brain, as distinguished from mere depravity, can be ascertained either with certainty or with a degree of probability sufficiently high to serve for practical guidance; and, secondly, is there any medical criterion of a degree or kind of lack of self control or of perverted judgment which ought to render the diseased person irresponsible for acts of a criminal character, or which ought to invalidate acts done in a civil capacity? The difficulties arising from the former question are those which are most frequently brought to the knowledge of the public, but those arising from the latter frequently involve cases of extreme hardship and injustice. Some seventy years ago, a specialist then well known in London established a hospital for the treatment of the maladies in which he was chiefly interested, and a few years later his eldest son, who at that time possessed about a thousand pounds, made a will bequeathing his property to his father's hospital. Shortly after the date of the will the testator became insane, and remained so for the rest of his life, the last twenty years of which were spent in a state of hopeless dementia. During the course of this period, property to a considerable amount devolved upon him from settlements and various other family sources, and he died a comparatively rich man. His early will governed the whole of the distribution of this subsequently acquired property, and diverted it from his nearest relatives and legitimate heirs to the hospital, the authorities of which would make no further concession than to allow a small annuity to the testator's brother, which they refused to continue to his widow. The impropriety of allowing an early will to dispose of property subsequently devolving upon a lunatic seems to require no demonstration; but the requirements of justice in the case of a crime committed by a person said to be insane are far less easily ascertained or disposed of. A murder of peculiar atrocity is almost certain to call forth a defence on the ground of insanity, and the very atrocity itself is often advanced as if it afforded evidence in support of the hypothesis. The newspapers are abundantly supplied with letters from correspondents who declaim against the iniquity of subjecting a madman to capital punishment, or, indeed, to any punishment, as well as with others from correspondents who maintain that, if a murderer be also a lunatic, the combination furnishes an additional justification for hanging him. A strong argument for the enforcement alike of criminal responsibility and of civil incapacity is furnished by the circumstance that the expectation of impunity is conducive to the commission of crime, and by the undoubted fact that many persons display, in their testamentary dispositions, insane hatreds or prejudices which they had been sufficiently cunning to keep in concealment during life. The whole subject is one which eminently calls for the combined labours of the physician and of the jurist, and is, moreover, one in which there is every hope that the lights of modern science may soon be brought into profitable application. The recent work of Dr. Leredde, of Paris, in the direction of showing the frequent dependence of tabes and of general paralysis upon syphilis, and their frequent curability by sufficiently early and energetic mercurial treatment, coupled with the frequent recognition of the syphilitic character of other brain lesions in the insane, opens out extremely hopeful prospects for the future, and may even go far to justify an expectation that the problem with which Sir John Tuke has set himself to deal may be rendered less pressing upon future generations than it is among ourselves. A recognition of the true place of syphilis in pathology would not only justify very stringent legislation, but would even put to silence, if not to shame, the persons who succeeded, a few years ago, in restoring it to full activity in the Army.

